# Supplementary material for: Content-rich biological network constructed by mining PubMed abstracts
Source: BMC Bioinformatics. 2004 Oct 8;5:147. doi: 10.1186/1471-2105-5-147 (PMC528731; doi:10.1186/1471-2105-5-147)
Supplement: Additional File 2 — The original results of the above study (non-essential files are deleted to keep the file size under the limit set by BMC bioinformatics). [file 1471-2105-5-147-S2.bz2 › chilibotAdditionalFile2/dip40/440ID9153236E10391/html/CCR7_CCL19.html]

 


 **CCR7** and **CCL19** 
  
Found 79 abstracts in PubMed, retrieved 40.  
 

 What does Google say? 
 PDF only 
| .edu only 

---

**Interactive relationship** (e.g. stimulation, inhibition, etc)

**Neutral relationship**- Cognate interaction of chemokine receptor  **CCR7**  on lymphocytes with its ligands  **CCL19**  and CCL21 expressed on high endothelial venules HEVs is essential for effective migration of T and B cells across HEVs into secondary lymphoid organs.  Ref: 11748287 J Exp Med, 2001
- **CCR7**  binds  **CCL19**  and CCL21, causing the highly activated DCs to be trapped in the peripheral tissue site.  Ref: 12414528 Am J Pathol, 2002
- and ELC  [ **CCL19** ] , which binds  **CCR7** , mediated significant chemotaxis of thymocytes.  Ref: 11507220 J Virol, 2001
- Neonatal appearance of circulating T cells was defective in  **CCL19**  neutralized mice as well as in  **CCR7**  deficient mice but not in CCL21 neutralized mice.  Ref: 11869682 Immunity, 2002
- In contrast, the majority of DC Lamp mature DCs in inflamed skin were totally negative for  **CCL19**  and were surrounded by  **CCR7**  memory type T cells.  Ref: 12474232 J Pathol, 2003
- Using immunohistochemistry, the majority of DC Lamp mature DCs in the T cell area of LNs expressed  **CCL19**  and were surrounded by  **CCR7**  naïve type lymphocytes, while CCL21 was expressed in reticular stromal cells and vascular endothelial cells.  Ref: 12474232 J Pathol, 2003
- ELC  [ **CCL19** ]  and SLC are potent agonists for  **CCR7** , a receptor of up most importance for the regulation of the homing and traffic of lymphocytes into and within secondary lymphoid tissues.  Ref: 11745346 Eur J Immunol, 2001
- Using LC type cells generated in vitro in the presence of transforming growth factor TGF beta1, we further found that tumor necrosis factor TNF alpha, as a prototype proinflammatory factor, and a variety of inflammatory stimuli and bacterial products, increase Langerin expression and Langerin dependent Birbeck granules formation in cell which nevertheless lack costimulatory molecules, DC LAMP CD208 and potent T cell stimulatory activity but express  **CCR7**  and respond to the lymph node homing chemokines  **CCL19**  and CCL21.  Ref: 12186835 J Exp Med, 2002
- We demonstrate that the CD4 CD25 CD62L splenocytes express  **CCR7**  at high levels and migrate toward secondary lymphoid tissue chemokine and ELC  [ **CCL19** ]  macrophage inflammatory protein 3beta, lymphoid chemokines.  Ref: 12193715 J Immunol, 2002
- The association among  **CCL19**  21 producing cells,  **CCR7**  expression, and mature DC accumulation is in line with the roles of these chemokines in mature  **CCR7**  DC homing to lymphocytic infiltrates.  Ref: 11994492 J Immunol, 2002
- The  **CCR7**  ligands, secondary lymphoid tissue chemokine SLC and Epstein Barr virus induced molecule 1 ligand chemokine  [ **CCL19** ]  ELC  [ **CCL19** ] , were recently recognized as key molecules in establishing functional microenvironments for the initiation of immune responses in secondary lymphoid tissue.  Ref: 11535317 Vaccine, 2001
- ELC  [ **CCL19** ]  induced a rapid time and concentration dependent internalization of  **CCR7**  and markedly decreased the ability of  **CCR7**  bearing cells to respond to a second stimulation.  Ref: 11745346 Eur J Immunol, 2001
- Furthermore, the expression of  **CCR7** , the receptor for both CCL21 and  **CCL19** , correlated with clinical lymphadenopathy, and blocking of  **CCR7**  inhibited CLL cell TEM.  Ref: 11929789 Blood, 2002
- The T cell chemokine receptor  **CCR7**  is internalized on stimulation with ELC  [ **CCL19** ] , but not with SLC.  Ref: 11745346 Eur J Immunol, 2001

**Non-interactive relationship** (e.g. studied together, co-existance, homology, etc.)

- Salmonella exposed DCs as well as mature DCs, but not immature DCs, were recruited towards the CC chemokines  **CCL19**  and CCL21, two ligands of  **CCR7** .  Ref: 12071677 Microb Pathog, 2002
- We studied the role of Rho kinase and extracellular signal regulated kinase ERK 2 in the polarization and migration of T lymphocytes in response to the  **CCR7**  ligands EBI1  [ **CCR7** ]  ligand chemokine ELC  [ **CCL19** ] .  Ref: 12729902 FEBS Lett, 2003
- We then focused on the expression of CCL20 CCR6 and  **CCL19**   **CCR7** , CCL21  **CCR7**  chemokine receptor complex, which control immature and mature DC migration respectively.  Ref: 11994492 J Immunol, 2002
- The binding to IP 10 could be inhibited by SLC but not by a non mac25 AGM binding chemokine, EBI1  [ **CCR7** ]  ligand chemokine ELC  [ **CCL19** ] .  Ref: 12847218 J Immunol, 2003
- Our results document that activation of human naive T cells and differentiation in Th1 or Th2 cells result in progressive down modulation of  **CCR7**  expression and  **CCL19**  responsiveness.  Ref: 11981813 Eur J Immunol, 2002
- Mesenchymal cells also secrete  **CCL19** , a ligand for  **CCR7** , yet it is not clear whether  **CCR7**  and  **CCL19**  are important for secondary lymphoid organ development.  Ref: 12732661 J Exp Med, 2003
- The U12 gene encoded a calcium mobilizing receptor for the EBI1  [ **CCR7** ]  ligand chemokine macrophage inflammatory protein 3beta ELC  [ **CCL19** ]  MIP 3beta but not for other chemokines, suggesting that the chemokine selectivity of the U12 gene product is distinct from that of the known mammalian chemokine receptors.  Ref: 12829849 J Virol< MedlineTA>J Virol, 2003
- The lymphocyte expressed chemokine receptor  **CCR7**  is playing an important role during this process, as its HEV presented ligands  **CCL19**  and CCL21 can trigger rapid integrin activation under flow in addition to inducing a chemotactic response, which may participate in transmigration and or interstitial migration.  Ref: 12393730 Blood, 2003
- These results indicate a  **CCL19**  and  **CCR7**  dependent pathway of thymic emigration, which represents a major pathway of neonatal T cell export.  Ref: 11869682 Immunity, 2002
- **CCR7**  expression, responsiveness to  **CCL19** , and L selectin alpha 4 beta 7 phenotype are coordinately regulated.  Ref: 12165486 J Immunol, 2002
- **CCR7**  and its ligands,  **CCL19**  and CCL21, are involved in lymphocyte recirculation through secondary lymphoid organs and additionally navigate lymphocytes into distinct tissue compartments.  Ref: 12023337 J Immunol, 2002
- Costimulation with PGE2 enhanced the expression of the  **CCL19**  CCL21 receptor  **CCR7**  on the cell surface of MoDCs when they were matured with soluble CD40 ligand or proinflammatory cytokines, but did not affect  **CCR7**  expression of polyI C stimulated MoDCs.  Ref: 12149218 Blood, 2002
- In the present study, using a murine spleen derived DC line, we analyzed effects of  **CCR7**  ligands,  **CCL19**  and CCL21, on dendritic morphology.  Ref: 12200351 Blood, 2002
- Transcripts for  **CCL19**  and CCL21 and their common receptor  **CCR7**  were induced in the CNS of mice undergoing relapsing remitting and chronic relapsing EAE.  Ref: 12580544 Brain Pathol, 2003
- In normal skin, on the other hand, no cells immunoreactive for  **CCL19** , CCL21, or  **CCR7**  were found.  Ref: 12474232 J Pathol, 2003
- The CC chemokine receptor CCR 7  [ **CCR7** ]  ligands CCL21 and  **CCL19**  were recently described as essential elements for establishing the microenvironment needed to initiate optimal immune responses in secondary lymphoid tissues.  Ref: 12890630 Virology, 2003
- Within 24h of local antigen priming, the expression of three chemokine receptor genes CCR3, CCR5, and  **CCR7**  and three chemokine ligand genes CCL12,  **CCL19** , and CCL25 were significantly upregulated.  Ref: 12705880 Biochem Biophys Res Commun, 2003
- The respective roles of the  **CCR7**  ligands  **CCL19**  and CCL21 in mediating migration are not fully defined, but chemotaxis to  **CCL19**  mediates Langerhans cell exit from the epidermis.  Ref: 11502161 Semin Immunol, 2001
- Another CC chemokine CCL21, which shares  **CCR7**  with  **CCL19**  but has a unique C terminal extension containing positively charged amino acids, failed to show involvement in thymic emigration.  Ref: 11869682 Immunity, 2002
- Here we show that antigen engaged B cells have increased expression of  **CCR7** , the receptor for the T zone chemokines  **CCL19**  and CCL21,.  Ref: 11882900 Nature, 2002
- In the present study, we examined the effects of  **CCL19**  and CCL21, two  **CCR7**  ligands, on endocytosis of fluorescein isothiocyanate FITC dextran by murine DCs.  Ref: 12609829 Blood< MedlineTA>Blood, 2002
- RT PCR analysis revealed expression of  **CCL19** , CCL21, and  **CCR7**  at high levels in LNs and at low levels in inflamed skin.  Ref: 12474232 J Pathol, 2003
- SIV infection altered the expression of chemokines  **CCL19**  MIP 3beta, CCL21 6Ckine, and CCL20 MIP 3alpha and of chemokine receptors  **CCR7**  and CCR6 that drive DC trafficking.  Ref: 12406887 Blood, 2003
- Plt mice, which lack expression of  **CCL19**  and CCL21 ser, both ligands for  **CCR7**  on HEVs, as well as  **CCR7**  deficient mice, have a defective cell migration and reduced homing of lymphocytes.  Ref: 11748287 J Exp Med, 2001
- In mice lacking lymphoid  **CCL19**  and CCL21 chemokines, or with B cells that lack  **CCR7** , antigen engagement fails to cause movement to the T zone.  Ref: 11882900 Nature, 2002
- Interestingly, a second  **CCR7**  ligand,  **CCL19** , triggered T cell sticking in cremaster muscle venules, but failed to induce extravasation in air pouches.  Ref: 12707342 J Immunol, 2003
- Thus, our data suggest an alternative G alpha i dependent,  **CCR7**   **CCL19**  CCL21 independent mechanism for lymphocyte homing through HEVs which is strongly augmented in the presence of FTY720.  Ref: 11748287 J Exp Med, 2001
- To analyse the nature of T cell mature DC interactions further in chronically inflamed skin and LNs, the present study focuses on another chemokine system, namely  **CCL19**  EBI1  [ **CCR7** ]  ligand chemokine.  Ref: 12474232 J Pathol, 2003
- **CCL19**  MIP 3beta, CCL20 MIP 3alpha, CCR6, and  **CCR7**  expression increased in lymph nodes during the early systemic burst of viral replication acute infection.  Ref: 12406887 Blood, 2003
